# Coping with the Forced Separation of Close Relationships during the COVID-19 Pandemic

**DOI:** 10.11621/pir.2022.0305

**Published:** 2022-09-30

**Authors:** Anna G. Samokhvalova, Mariia V. Saporovskaia, Svetlana A. Khazova, Elena V. Tikhomirova, Natalia S. Shipova

**Affiliations:** a Kostroma State University, Kostroma, Russia

**Keywords:** Forced separation, close relationship, relationship stresses, coping behavior

## Abstract

**Background:**

Issues regarding close relations became especially prevalent within the context of the pandemic, due to the forced separation of these relationships. This is now seen as a significant stressor that influences emotions and subjective perceptions of the relationship.

**Objective:**

The current study aims to investigate the specifics of coping with the forced separation of partners with varying types of closeness.

**Design:**

The study was carried out with quantitative and qualitative methods. The sample included 43 women and 42 men (aged 21–47); all were involuntarily separated from their partners by impacts of the pandemic.

**Results:**

Three scenarios of romantic relationships were identified based on a balance of love components (intimacy, passion, commitment) and prevalent feelings (closeness or distance). The authors concluded that certain coping strategies (positive reassessment, acceptance, distraction) can help the individual to cope with the separation, but do not contribute to the closeness of the partners and the strengthening of the relationship. Coping primarily through active support was typical for partners with intense and balanced feelings based on closeness (Scenario 3). Ambivalent feelings toward a partner (Scenario 1) were associated with passive coping, which increased the risk of detachment. The moderate manifestation of balanced love components and closeness (Scenario 2) focused on acceptance of the situation, positive reassessing, and distraction, all of which reduce the stress of separation, but cause anxiety regarding the future of the relationship.

**Conclusion:**

The type of close relationship has been found to correlate to the coping strategy adopted by the couple following forced separation during the COVID-19 pandemic. However, further studies are required to investigate separation in various social contexts, not only in isolation due to the pandemic, as well as its connection to psychological well-being.

## Introduction

### Close relationships in the contemporary social and cultural context

Contemporary challenges (large-scale migration processes, the rapid development of cyberspace, globalization, digitalization, pandemics, etc.) are leading to the transformation of close relationships, including family relations, from conservative and conventional (familialism) (Revillard, 2006) to post-modern (McDaniel, 2015; Muniruzzaman, 2017; Accordini et al., 2018; Harris et al., 2020; Luetke et al., 2020). The latter involve a wide range of partnership forms, including amorphous relations with an unstable system of rules and an absence of rigid norms and the algorithms with which to build them (Henslin, 2004).

Despite transformational processes, close relationships remain an important social resource for the development, psychological well-being and stress resistance of a person in the context of modern life (Cavallo et al., 2014). On the one hand, they have a positive effect on the psychological and physical state of the individual through the satisfaction of his needs for affection, love, acceptance, care, trust, support, a sense of happiness, and the accumulation of positive life experience (Kawamichi et al., 2016)

On the other hand, close relationships are sensitive to the broader social context. Their preservation, development, and stabilization in conditions of high social tension and uncertainty require additional resources, such as motivation , strong-will, and skills for solving relationship problems and coping with external stressors.

We understand close relationships as a special kind of interpersonal relationship that is both meaningful, aimed at satisfying the human need for love and belonging. Close relationships are based on affiliative feelings and attachment to a partner, characterized by intimacy, informality, significance, long-term co-existence, and emotional depth (Aron, 1992; Sternberg, 1996; Kryukova et al., 2019, Saporovskaya, 2021).

The identification of common categorical features of close relationships (mutual attachment to the other; the bringing together of emotions and feelings; psychological distance; trust; value-semantic unity of involvement) forms the basis for the study of romantic and marital relationships.

The construct of relationship quality is of particular importance. It is the quality of close relationships that largely determines the well-being of a person (a sense of fullness of being, health, joy, or, conversely, alienation, loneliness, unhappiness) and the success/failure of group functioning (for example, a family). However, the quality of close relationships depends on a large number of factors: personality and its self-awareness (identity), emotional intelligence, patterns of interaction with other people, and the support and responsiveness of a partner. A special place in this system is occupied by an external factor: the socio-cultural context. This includes constant distraction and life changes that cause tension or uncertainty and can act as stressors.

The rapidly changing world, the increase in its complexity, information overload, lack of security and the impossibility of predicting the future has become a meta-trend in human life, society and the socio-cultural context of close relationships.

### The COVID-19 pandemic and forced separation of close relationships

The problem of stress and coping in close relationships has gained particular importance against the backdrop of the COVID-19 pandemic. Restriction of freedom of movement, narrowing of interpersonal contacts, the transition to a remote work format, limited eventfulness of everyday life, misinformation, financial losses, and stigmatization became a source of additional stress in close relationships (Brooks et al., 2020; Everett et al., 2020; Yip et al., 2020). The quality of relationships was strongly influenced by the fear of infection, forced self-isolation and the uncertainty of the future, associated with an increase in anxiety (Brooks et al., 2020; Everett et al., 2020; Qiu et al., 2020).

In general, the pandemic has become associated in the cognitive field with the problem of separation in close relationships. Due to the risk of infection and the introduction of social distancing norms, interpersonal relationships, even within a couple or family, have undergone great changes (Bowen, 2021; Jones et al., 2021; Pietromonaco et al., 2022; Montanaro et al., 2022).

On the one hand, under the condition of living together, couples and families found themselves cut off , isolated from the wider system of social ties. Researchers emphasize the ambiguity of the consequences of such a situation in the following:

- Convergence, integration of resources, formation of an effective dyadic coping system, mutual enrichment, reduction of anxiety associated with potential loneliness.
- Satiety with relationships, growing conflicts, loss of interest in a partner, accumulation of irritation, breakup of relationships.
- Manifestation of destructive tendencies in a couple, such as violence, psychological pressure, sexual dysfunction (Luetke et al., 2020; Yang et al., 2020; Candel et al., 2021).

On the other hand, especially at the beginning of the pandemic, many partners and families were separated geographically, which contributed to the search for ways to continue communication and maintain closeness at a distance. At this time, internet communication and other means of mediated interaction were intensified.

In this article, our attention is focused on the forced separation of close relationships, which, in the context of the pandemic, has acquired an uncertain character in terms of the duration of the separation. E.W. Burgess pointed to the importance of long-term separation of family members among the factors that can cause a family crisis, along with economic depression, family shame, and unjustified expectations (Burgess, 1956). At the same time, the long-term spatial separation of the subjects of close relationships (living in different cities and countries, the impossibility of crossing borders, the rotational method of professional activity with an extension for an unlimited period of stay at the place of work, distance learning in educational institutions, and self-isolation of people over 65), as well as uncertainty regarding the point of reunification during the COVID-19 pandemic became part of everyday life for a large number of people around the world. This was associated with the perceived need to solve important personal and social problems. The pandemic became a test for people and their feelings for each other (Sweeny et al., 2020; Qiu et al., 2020).

Attempts have already been made to understand the phenomenon of forced temporary separation due to various other factors in modern human life, namely population migration as a result of a difficult geopolitical situations (Silver, 2006; Moskal, 2016; Martin, 2017); the possibility of building a career and getting a higher salary in other countries (Kariuki, 2014); service in the army and other enforcement bodies (Holland, 1997); and now the need to minimize contact during the COVID-19 pandemic (Montauk et al., 2020; Goldberg et al., 2021).

The main consequence of separation is a change in the microsocial situation, which can lead to both stabilization and destabilization of the system of close dyadic relations. Researchers note possible negative consequences such as a weakening of trust, increased anxiety, the development of relationship toxicity, interpersonal addiction, communicative discomfort during the transition from personal to indirect communication and vice versa, ruin of sexual relations, and reduced fertility (Hong, 2020; Nie, 2020; Ojeda, 2020). At the same time, scientists are inclined to believe that separation can also become a facilitator of positive changes in relationships.

What makes separation a destabilizing or stabilizing factor?

The analysis of studies of this problem enabled us to identify three main factors:

1. Personal meaning and cognitive assessment of the situation of separation.
2. The quality of close relationships prior to separation.
3. Coping resources and the specifics of partners’ coping with the situation of forced separation from each other.

Firstly, the dynamics of the relationship and the propensity of separation to cause stress depend on the personal perception, cognitive evaluation and implied meaning of the situation. Is separation a threat to welfare and stability in the relationship? Is separation a part of everyday reality to which the partners are well adapted through the carefully developed system of individual and dyadic coping?

If the separation is stressful, then this can negatively affect the perception of a partner and the interpretation of information about the quality of the relationship (Karney, 2004). Separation from a partner can also create an environment for cheating and infidelity (Dowdle, 2016). Th us, the situation of separation can become a facilitator of other stresses in close relationships of partners.

The most sensitive to the situation of separation are such variables as: self-perception and self-disclosure (Horn et al., 1997); characteristics and patterns of communication (Dainton et al., 2009); cognitive assessment (for example, idealization) of relationships (Acosta-Rodas et al., 2021); obligations (Reader, 2003); closeness (Holmes, 2010).

One of the important positive effects of separation can be the experience of being bored, which can motivate partners to strengthen the relationship. When people miss each other, they try to be active in communication, maintain their relationship and avoid behavior that threatens the well-being of the relationship e.g., infidelity (Le et al., 2010).

To understand the complexity of separation, its variety and polymodality, we focused on a long-term forced separation in close dyadic relationships resulting from the COVID-19 restrictions. This separation has been found to be accompanied by a number of negative psychological effects; increased inner tension, increase in personal and social loads, negative emotional reactions, the feeling of subjective loneliness, jealousy, and fear for the future of the relationship (Démurger, 2015). Therefore, it needs studying separately. The situation of forced long-term separation is characterized by uncertain duration and postponed reunions. Hence, this lack of control tends to increase the partners’ stress load. (Gallagher et al., 2014).

Secondly, the dynamics of the relationship during separation are related to the quality of the relationship prior to separation. The actualization of the experience is a major resource to cope with separation and associated stress. The quality of the relationship (perceived value of the partner, distance/closeness, level of trust) before separation will influence the cognitive evaluation of the stress-inducing situation and the ability to cope with accompanying negative states (fear, anxiety, emotional exhaustion, mistrust, jealousy, loneliness, etc.). It will also predict the dynamics of the relationship aft er the separation in terms of relative closeness/distance.

The factor theory by R. Sternberg identifies three basic features of love: intimacy (sexual closeness as an emotional feature), passion (the attractiveness of a partner as a motivational feature), and commitment (the behavioural aspect, reflecting care for the partner and for the development of the relationship) (Sternberg, 1996). The ratio of these features (degree and balance) does not only define the specifics of the relationship, but also estimates the intensity of any stressful influence, including separation (Sternberg et al., 2001). The quality of the relationship is manifested in the need (or its absence) to maintain and strengthen the relationship, its subjective value, the range of feelings (bonding or distancing) and the emotional states that emerge during separation.

The initial perceived emotional responsiveness of partners and assessment of closeness play an important role as they can minimize the destabilizing effect of external stressors on relationships (Balzarini et al., 2020).

Negative previous experience of relationships and individual and group vulnerability enhance destructive dyadic processes and reduce the quality of relationships in a couple, making them even more “fragile” (Karney et al., 2021).

Thirdly, the system of coping resources and strategies used by close partners becomes extremely important in the context under investigation. Standing behind intense and oft en negative human responses to stress is an inadequate development of coping mechanisms (Uchino, 2009, Cohen, 2004). Therefore, coping in the relationship is actualized within problems related to the dyadic functioning (Bodenmann et al., 2010). Th ere is evidence that separation from a partner and positive changes in the quality of the relationship are possible if partners support each other and meet regularly online, provided these meetings are not just a formal exchange of information, but emotional communication and an experience of closeness (Hong, 2020). It means that relationship-oriented coping performs a regulatory function; cognitive and behavioural efforts to establish and maintain connection (Bodenmann et al., 2010).

It should be noted that in most studies, attention is focused on changes in the qualitative parameters of close relationships during separation, and the interaction of factors of its cognitive assessment. In this way, coping with the separation and the quality of the relationship does not fall into the focus of attention of researchers. But it is precisely the relationship of these three factors that determines the positive or negative dynamics of close relationships during separation.

Th us, when defining a forced long-term separation as stress, it must be emphasized that it is not necessarily followed by regress, destabilization, or the deterioration of the relationship. The dynamics are related to both the quality (type) of close (romantic) relationship and the specifics of the perception of separation, as well as the specifics of coping.

The research question: what are the coping specifics during the separation of partners with different types of close relationships?

## Methods

### Participants

The research aimed to study the specifics of coping behavior in the context of a longterm forced separation with different types of close relationships.

The study was carried out in the cities of the Central Federal District of the Russian Federation (Kostroma, Yaroslavl, Ivanovo, Vladimir) and Yamalo-Nenets autonomous area of the Russian Federation during the period of lockdown in place due to the COVID-19 pandemic from January 15 — May 15, 2021.

The participants were adult men and women who found themselves forcibly separated from their partner for a long period (more than one month).

The sample included 85 adults in total:

- Group 1: 42 men aged 22–45 (M=35.74, SD=3.78); 69% of whom were married and had their own children born in marriage, 31% were in a close relationship with a female partner; the relationship duration varied from 2 to 21 years; 88,1% had a degree, 11,9% had a vocational education. Participants permanently lived in cities of the Central Federal District (30,9%), North-Western District (26,2%), Privolzhsky District (23,8%) and Siberian District (19,1%); 100% of the respondents at the time of the study worked on a rotational basis in the regions of the Far North on natural gas fields (Yamalo-Nenets Autonomous Region);
- Group 2: 43 women aged 21–47 (M=33.86, SD=6.95); 86% of whom were married and had their own children born in marriage, 14% were in a close relationship with a male partner; the relationship duration varied from 1 year to 15 years; 74,4% had a degree, 25,6% had a vocational education. Participants permanently lived in the cities of the Central Federal District of the Russian Federation (Kostroma — 48,8%,Yaroslavl — 34,9%, Ivanovo — 11,7%, Vladimir — 4,6%); 100% of the female sample were employed in the education, healthcare and social spheres and were partners of male shift workers who, due to the closure of borders between regions during the pandemic, were forced to remain indefinitely at the workplace.

The samples were not interconnected i.e., the respondents from Group 1 were not romantically related to those from Group 2.

All respondents had the experience of a fixed-duration, voluntary separation from a partner for a period of one month, which men spent on a work shift . During the pandemic, the duration of separation was indefinitely long, since the men, having left for work, were not able to return home and did not know when they could be reunited with their spouse/lover. At the time of the study, the period of separation was more than 2 months. Th us, separation among respondents began as short-term voluntary with a fixed duration of 1 month, but due to the pandemic, it had become long-term and forced, with an indefinite end date (in fact, the period of separation during the pandemic was extended to 5-6 months instead of 1 month).

### Procedure

The study was conducted in an online format. Links to the survey were posted on social networks, on the personal pages of the team of authors, and in the organizational groups of the university. Participation in the study was voluntary, taking into account the principles of confidentiality and environmental friendliness, the respondents were informed about the purpose of the study, about their rights, and were warned that the results would be used exclusively for scientific interests and only in a generalized form

### Questionnaires

The research was conducted using the following tools:

1. The questionnaire to collect social and demographic information (gender, age, education, salary level, length of relationship, place of residence, place of work, duration of shift / separation, length of work on a rotational basis, age of spouse / romantic partner, the frequency of indirect contacts with a partner, the presence / absence of sexual relations with a partner).
2. Scales of self-esteem where the respondents were asked to rate how happy they are in their relationship on a ten-point Likert scale, and to indicate the degree of mutual trust, the degree of intimacy, the degree of anxiety when separated from a partner.
3. Triangular Love Scale, R.J. Sternberg, 1986/1997. 45 items, 3 scales; 9-point Li kert scale) (1 — absolutely not, 9 — absolutely). The research results from an American sample by R. Sternberg (1986) are as follows (n=101, Cronbach’s alpha (*а*) intimacy 0.94, passion 0.94, commitment 0.97). The research results from a Russian sample by O. Yekimchik (2011) are as follows: (n= 256, Cronbach’s alpha (*a*) 0.94/0.93/0.95).
4. Profile of feelings in the relationship questionnaire by L.V. Kulikov (2003). The questionnaire focuses on two types of interaction: bonding feelings (9 feelings) and distancing feelings (9 feelings). Likert scale (1–7): 1 — a very weak feeling, 7 — a very strong feeling.
5. Brief Cope by C.S. Carver (Carver, 1997): 28 items, 14 conceptually differentiable coping reactions, n=126, Cronbach’s alpha (*а*) : active coping 0.68 / planning 0.7З / positive reframing 0.64 / acceptance 0.57/ humor 0.73/ religion 0.82/ using emotional support 0.71 / using instrumental support 0.64 / self-distraction 0.71 / denial 0.54 / venting 0.5 / substance 0.9/ behavioral disengagement 0.65 / self-blame 0.69. The instructions asked the respondents to imagine their particular situation of separation from the romantic partner.
6. To study the peculiarities of the separation perception the respondents were asked to complete the sentence: “Separation from my partner is ... to me”, and answer the direct question: “What are my worries about the separation?”.

The collected data were processed with the soft ware pack SPSS Statistics 22.0.

The preliminary stage involved the assessment of the normality of criteria distribution through calculations in descriptive statistics by using Kolmogorov-Smirnov consent criteria (Kolmogorov-Smirnov Test). To identify the types of romantic relationships in adults we used cluster analysis by the method of K-means. To define the predictions of coping strategies we used regression analysis by step-by-step Ridge Regression. The differences between the groups were assessed using the Kruskal-Wallis H-test and Fisher’s F-test.

## Results

The first empirical aim of the research was the identification of the relationship types. They were identified using K-means clustering analysis, based on the following variables: love components (intimacy/closeness, passion, commitment/promises) and dominant feelings (bonding or distancing) (see *Table 1*).

**Table 1:** Results of Cluster Analysis

Variables	Cluster No. 1 (17 respondents)	Cluster No. 2 (20 respondents)	Cluster No. 3 (48 respondents)
Intimacy / Closeness	6.83	6.16	8.12
Passion	6.63	5.63	7.32
Commitment/Promises	6.69	5.97	7.78
Bonding	52.82	33.30	51.19
Distancing	31.29	8.45	7.35

*Note. VP = Valid Percentage. CP = Cumulative Percentage.*

The first cluster included 17 respondents: 8 men aged 20–25 (M = 26.5, SD = 5.15) and 9 women aged 16–29 (M = 20.44, SD = 3.97).

The second cluster included 20 respondents: 9 men aged 20–29 (M = 25.55, SD = 2.96) and 12 women aged 19–47 (M = 27.33, SD = 7.79).

The third cluster included 48 respondents: 25 men aged 19–34 (M = 25.56, SD = 3.67) and 22 women aged 18–40 (M = 23.36, SD = 6.84).

The analysis of the differences in the manifested degree of love components revealed higher scores for all indicators in the third cluster: Intimacy (F = 14.76, p ≤ 0.000), Passion (F = 7.89, p ≤ 0.000), Commitment (F = 9.77, p ≤ 0.000)

However, it is worth mentioning that all values lie in the middle, indicating low intensity during a long-term separation (Sternberg, 1996).

As for the differences in the feelings leading to closeness or distancing, all the three clusters differ considerably. Th us, bonding feelings dominate among the respondents from the first and third clusters in comparison with the second (F = 64.08, p ≤ 0.000). At the same time, distancing feelings are more clearly manifested in the first cluster compared to the other two (F = 64.76, p ≤ 0.000).

**Table 2:** Means (M) and Standard Deviations (SD) for Coping Strategies

Coping Strategy	Cluster No. 1		Cluster No. 2		Cluster No. 3	
	M	SD	M	SD	M	SD
Self-distraction	5.88	1.83	5.70	1.56	5.73	1.59
Active coping	5.06	1.82	5.10	1.45	6.52	1.25
Denial	3.88	1.62	2.65	1.09	2.98	1.47
Substance use	3.82	1.78	2.40	0.99	2.58	1.30
Search / use of emotional support	5.00	1.50	4.30	1.89	5.04	1.77
Search / use of instrumental support	4.29	1.49	4.10	1.80	4.23	1.98
Avoidance	3.76	1.48	2.40	1.35	2.65	1.25
Venting of emotions	4.53	1.62	3.70	1.75	3.67	1.59
Positive reframing	5.12	1.65	5.75	1.41	6.15	1.71
Planning	5.24	1.56	4.55	1.54	5.96	1.56
Humor	4.65	1.73	5.40	1.67	4.81	1.90
Acceptance	5.24	2.11	6.55	1.47	6.50	1.49
Religion/faith	4.18	1.88	2.65	1.46	2.73	1.12
Self-blame	4.41	1.73	2.85	1.27	3.02	1.23

The clusters were matched according to coping strategies. Interestingly, in all three clusters, the most used strategies were acceptance and positive reframing. However, in addition to this, in the first cluster, the most frequent methods were self-distraction (M = 5.88, SD = 1.83) and planning (M = 5.23, SD = 1.56). In the second cluster, the preferred strategies included self-distraction (M = 5.70, SD = 1.55) and humor (M = 5.40, SD = 1.66). The third cluster demonstrated strategies of active coping (M = 6.52, SD = 1.46) and planning (M = 5.95, SD = 1.55).

Differences were identified for six out of fourteen coping strategies. Those least focused on actively coping with the separation situation (H = 15.9, p = 0.00) and planning actions were the respondents of the first and second clusters (H = 14.2, p = 0.00). At the same time, the respondents of the first cluster, more than in the other two clusters, expressed such coping strategies as the use of psychoactive substances (H = 14.85, p = 0.00), denial (H = 6.4, p = 0.04), avoidance (H = 17.2, p = 0.00), religion / faith (H = 13.6, p = 0.001), and self-blame (H = 11.43, p = 0.003).

The results of regression analysis indicated an insignificant role of the components of love (intimacy, passion, and commitment) and bonding or distancing feelings in predicting strategies for coping with the stress of separation.

In the first cluster, their cumulative influence allows predicting only two strategies out of fourteen. However, the determination coefficients indicate a high predictive potential of the model: denial (F = 3.35, p < 0.044; R = 0.77, R2 = 0.60) and planning (F = 6.09, p < 0.006, R = 0.85, R2 = 0.73). In the first case, the greatest contribution is made by passion (β = 0.95, p ≤ 0.011). In the second case, a positive effect is provided by commitments / promises (β = 1.27, p ≤ 0.017), which contribute to systematic coping actions, negative – intimacy (β = -1.06, p ≤ 0.047), passion (β = –0.69, p ≤ 0.019) and distancing feelings (β = -0.63, p ≤ 0.003), which reduces the possibility of planning a solution to the problem. In the second cluster, only two strategies can be predicted with a high degree of probability by the predictors we have identified. Th us, more than 65% of the variance of the “acceptance” variable is predicted (F = 5.41, p < 0.005, R = 0.812, R2 = 0.658) with a significant contribution of the “intimacy / closeness” variable (β = 1.05, p ≤ 0.012) and 53% of the variance of the variable “self-blame” (F = 3.20, p < 0.039, R = 0.730, R2 = 0.533) on the part of the variable “commitment/promises” (β = 1.41, p ≤ 0.005) and “distancing” (β = 0.052, p ≤ 0.015). In the third cluster, the dependence of 36% of the variance of the variable “venting of emotions” (F = 4.68, p < 0.001, R = 0.59, R2 = 0.36) on independent variables was established, with the “commitment/promises” indicator (β = –0.850, p ≤ 0.001) playing the leading role.

## Discussion

In this research we verified the hypothesis that partners with different types of close relationships have different strategies of coping with separation.

The results enabled us to describe the specifics of romantic relationship types that manifested themselves in the degree and balance (or harmony) of love and passion. We also described the features of forced separation and the specifics of coping by partners with different types of romantic relationships.

The respondents from the first cluster were characterized according to the moderate manifestation and balance of love components alongside ambivalent feelings (both bonding and distancing feelings were present). Partners within these relationships can experience unity, a sense of value, friendliness, and respect (bonding feelings), but off ense, loneliness, and guilt (distancing feelings) may also be present. 74% of respondents with this type of close relationships perceived forced separation negatively: feelings of sadness, loneliness, hopelessness, and proneness to conflict were prevalent. This is oft en due to drastic changes to the intimate life. The American sample also showed that, since the spread of coronavirus and the associated social distancing measures in the, Americans have experienced escalations in conflict in their romantic partnerships, which were associated with changes to their intimate and sexual lives (Luetke et al., 2020). 26% of participants, however, consider separation as a chance to test mutual feelings, have a rest from each other, anticipate a pleasant reunion, and intensify romantic feelings. It is worth mentioning that 52% of this cluster were not sure whether the reunion would be possible (“I am worried whether we can be together aft er separation”). We can refer to this cluster as *moderately balanced love with an ambivalence about feelings of closeness.*

Coping with separation was found to be the contradictory phenomenon in all the three clusters. Some partners accepted the situation and tried to draw positives from it, while others denied reality and refused to believe in it. They distracted themselves from the situation by various activities (walking, meditation, sport) and by working harder, seeking emotional support from other people, and/or turning to religion. They were less oriented (in comparison to other respondents) towards active coping strategies; they oft en turned to substance (alcohol) abuse and were prone to self-blame and self-criticism. They were stuck at the planning stage of their coping strategy, but avoided taking action. Their romantic feelings caused a refusal to believe that the situation was serious, making planned actions uncertain and conflicting. On the one hand, obligations to their partner triggered them to start planning possible steps. On the other hand, feelings of both closeness and distancing prevented their employment of active coping strategies.

The respondents of the second cluster had an average degree and balance of the components of love (intimacy, passion, commitment), with an average degree of intensity of bonding feelings. Feelings of distancing (shame, resentment, contempt, envy) were presented at a low level. Th is, in our opinion, indicates a balanced state of love with a balanced, not intense emotional background.

57% of respondents with this type of close relationship perceived forced separation from a partner as distancing, loneliness, and anxiety. At the same time, 43% of respondents perceived the separation as a worthwhile experience, an opportunity to test feelings, a pleasant and exciting anticipation, or a neutral situation that does not change the quality of everyday life. At the same time, more than half of the respondents (61%) were worried about a possible breakdown of their relationships (“I’m not sure that he will return to me”; “I am worried that we will break the habit of each other and will not be able to be together”). We can refer to this cluster as *balanced love with moderately close feelings.*

Such a low intensity of feelings leads to a kind of coping, which can also be called “balanced”, oriented at the situation: the most pronounced here is the strategy of acceptance. This is predicated by the following: the emotional side of the relationship, primarily by the level of intimacy (1 R); positive reassessment of the situation and a humor-based attitude (2 R); distraction from negative experiences and thoughts by a variety of activities (3 R). Interestingly, high levels of obligation towards a partner and feelings of emotional distancing or withdrawal oft en led to self-blame: it seems that obligations to maintain love when feeling lonely or hopeless lead to the perception of oneself as the culprit.

In the third cluster, the respondents had high values for all the components of love (intimacy, passion, commitment), with a predominance of feelings of closeness (feelings of distance and withdrawal or detachment were minimally presented, and 15% of respondents did not note distancing feelings at all). This indicates a high intensity of feelings for a partner, a high level of rapprochement, and unity. 42% of respondents in this cluster noted the positive aspects of separation: the joy of each meeting, the value of intimacy, and confidence in a partner. 27% believed that longing for a partner makes it possible to understand the value of the relationship. Only 31% of participants in this cluster perceived this situation as pain, longing, or sadness. Only 21% were worried about the likelihood of the breaking off of relations aft er the forced separation, and 55% noted an improvement in relations during separation (“we began to love each other more,” “I didn’t know before how caring and gentle he was”, “how he cares about me and understands me”). It correlates with the results of the study that showed that the partner’s perceived responsiveness buffers people from lower relationship quality associated with COVID-related stressors (Balzarini et al., 2020). We can refer to this cluster as *intensely harmonious love with a predominance of close feelings.*

Coping strategies by partners with this type of close relationship was more active. A vibrant sensory background, along with accepting and appreciating the separation experience, led to proactive action and planning efforts. The respondents were not inclined to drink alcohol or avoid the situation, deny its significance or resort to religion. More intense love feelings forced them to express negative feelings about separation more strongly, to actively “throw them out”, to strive to get rid of the emotional load.

It should be mentioned that the respondents of all three clusters noted a preference for separation strategies of acceptance and positive reframing, which can be explained by the peculiarities of the situation, characterized by a low degree of its control, implying the uselessness of using active coping strategies. It is also important to have a previous experience of separation from a romantic partner (previously, separation was short-lived and planned). Forced separation for an indefinite period is poorly amenable to solution (depending on the epidemiological situation in the world) and planning (it is impossible to predict the timing of its completion).

## Conclusion

Th us, forced long-term separation is a stressor to close relationships. Spatial separation of partners, the lack of a sense of subjective control over the situation, and the absence of a stable system of dyadic coping, as well as uncertainty and isolation, result in loneliness, longing, sadness, and possible distancing (or alienation of the partner). Separation is both an external stress, based on the situational context of an intimate relationship, and an internal stressor, since the state of a relationship at the time of separation (the degree of feelings of love and closeness, and beliefs about the quality of the relationship), and the effectiveness of coping strategies can determine the level of stress during the separation and influence the future of the relationship aft er separation.

Coping strategies become a reliable indicator of the quality of close romantic relationships. Acceptance, avoidance, distraction, positive reframing, and action planning help to cope with separation on an individual level, but do not contribute to the bonding of partners, nor the maintaining and strengthening of the relationship. It is important that coping itself depends on the quality of close relationships (the ratio of intimacy, passion, and commitment, and bonding or distancing feelings), which gives rise to its variability. However, coping focused on emotional and cognitive support, that allows the maintaining of a close connection with a partner during stressful events, is rarely actualized. This largely explains the fragility of romantic relationships and increases the destabilization.

## Limitations

The findings showed that the type of close relationship, and the specificity of its perception are associated with coping strategies in the situation of stressful forced separation during the spread of COVID-19. However, it cannot be argued that the identified trends are rigidly determined by the situational context of isolation and deprivation during the pandemic. This requires further verification. In addition, this study has a number of limitations in the generalization of the conclusions: the relatively small size and inhomogeneity of the male and female groups of respondents, the lack of control measurements (e.g., before or aft er the pandemic), the lack of comparison with other types of separation (e.g., forced and short-term; or voluntary long-term). The findings confirm the need to study the situation of separation in different social contexts and establish a connection between the experience of this situation and psychological well-being.
